# Clinical Application of Detecting COVID-19 Risks: A Natural Language Processing Approach

**DOI:** 10.3390/v14122761

**Published:** 2022-12-11

**Authors:** Syed Raza Bashir, Shaina Raza, Veysel Kocaman, Urooj Qamar

**Affiliations:** 1 Department of Computer Science, Toronto Metropolitan University, Toronto, ON M5B 2K3, Canada; 2 Dalla Lana School of Public Health, University of Toronto, Toronto, ON M5T 3M7, Canada; 3 Data Science, John Snow Labs Inc., Lewes, DE 19958, USA; 4 Institute of Business & Information Technology, University of the Punjab, Lahore 54590, Pakistan

**Keywords:** COVID-19, named entities, clinical, non-clinical, social determinants of health, pipeline, de-identification

## Abstract

The clinical application of detecting COVID-19 factors is a challenging task. The existing named entity recognition models are usually trained on a limited set of named entities. Besides clinical, the non-clinical factors, such as social determinant of health (SDoH), are also important to study the infectious disease. In this paper, we propose a generalizable machine learning approach that improves on previous efforts by recognizing a large number of clinical risk factors and SDoH. The novelty of the proposed method lies in the subtle combination of a number of deep neural networks, including the BiLSTM-CNN-CRF method and a transformer-based embedding layer. Experimental results on a cohort of COVID-19 data prepared from PubMed articles show the superiority of the proposed approach. When compared to other methods, the proposed approach achieves a performance gain of about 1–5% in terms of macro- and micro-average F1 scores. Clinical practitioners and researchers can use this approach to obtain accurate information regarding clinical risks and SDoH factors, and use this pipeline as a tool to end the pandemic or to prepare for future pandemics.

## 1. Background

The COVID-19 pandemic (coronavirus disease 2019) has had a significant impact on society, due to the severity of the disease and the slow implementation of public health measures [1]. Many of these challenges stem from the information overload problem, which is exacerbated by the growing understanding of the disease and a plethora of literature on the subject [2]. COVID-19 Open Research Dataset (CORD19) [3] and LitCOVID [4] are among the pioneering data sources made available by the research community to aid collaboration between the computing community and the many stakeholders in the COVID-19 pandemic. These data sources contain hundreds of thousands of articles, and new articles are added regularly [1,5]. In its current state, it is difficult for researchers, clinical experts, and practitioners to obtain up-to-date information on the most recent findings.

To study the risk factors associated with COVID-19, government organizations and health sectors can always arrange for human resources to convert the pools of information from the literature into a structured format. However, by the time this data is made accessible to the research community, much of the earlier information is outdated. Natural Language Processing (NLP), a branch of artificial intelligence (AI), allows automated processing and analysis of unstructured texts, such as extracting key information and representing it in a structured format appropriate for computational analysis [6].

The goal of this research is to study the clinical factors, such as disease, drugs, treatments, procedures, and non-clinical factors, such as social determinants of health (SDoH) from the biomedical texts. In terms of methodology, we employ the named entity recognition (NER) [7] task of NLP to extract the biomedical factors from the free texts.

Despite being highly useful, the state-of-the-art work [8,9,10,11] in biomedical NER is primarily focused on a small number of entities (disease, chemicals, genes, etc.). There are numerous other clinical factors to consider, such as diagnosis, therapies, medical concepts, risks, and vital signs, as well as non-clinical factors such as SDoH. Extracting these biomedical entities (clinical and non-clinical) is important to study the predictors of COVID-19, which is a motivation for this research. Usually, scientific texts, such as clinical reports, medical notes, and Electronic Health Records (EHR) consist of sensitive patient information that must be de-identified. In this work, we also preserve patients’ private information through the data obfuscation process.

We have extended our previous work [12] in this paper and performed a more detailed analysis. We also fine-tune a transformer module to create the task-specific embeddings in this work. Our contributions are listed below:

- We develop a biomedical NER pipeline to identify clinical as well as non-clinical named entities from the COVID-19 texts. We attempt to consolidate and explain data science best practices through this pipeline, with numerous convenient features that can be used as it is or as a starting point for further customization and improvement.
- We develop a new dataset by curating a large number of scientific publications and case reports on COVID-19, and we scientifically parse the text from these scientific articles and prepare a dataset from it. We annotate a part of this dataset on biomedical-named entities to prepare a gold-standard dataset to train the NER pipeline. A portion of the gold-standard dataset is also reserved as a test set.
- We de-identify the patients’ personal information after identifying the named entities, thus adhering to the Health Insurance Portability and Accountability Act (HIPAA) [13].
- We demonstrate the efficacy and utility of this pipeline by comparing it with the state-of-the-art methods on public benchmark datasets. We also show the key findings related to COVID-19 in the analysis.

## 2. Previous Work

Named Entity Recognition (NER) is the task of identifying a named entity (a real-world object or concept) in unstructured text and then classifying the entity into a standard category [7]. In the field of biomedicine, NER is the task of identifying entities such as genes, diseases, chemicals, and proteins [11]. Several datasets are proposed for the NER task. These datasets are prepared usually in the CONLL-2003 format [14], a prototypical format for NER datasets. Many machine learning and deep learning based NER models have also been released in the past few years. Below, we summarize the benchmark datasets and methods used for NER in Table 1:

According to the Healthy People 2030 initiative, SDoHs related to population health [31] have a major impact on people’s health, well-being, and quality of life, and are related to health outcomes; this is a rather underexplored area of research in biomedicine and clinical research. In this work, we mention some SDOH in our dataset.

In a 2016 survey, nearly 95% of eligible hospitals in the United States use EHRs [32], with that figure expected to rise in these years. The standard EHRs contain 18 categories of critical private information about patients (e.g., name, age, and address), which must be de-identified before they are made public, as required by HIPPA [33]. For the de-identification purpose, researchers used a variety of methods, including rule-based, machine learning-based, and hybrid [34]. The CRF models [35], and Structured Support Vector (SVM) [36] are some of the commonly used models for NER and de-identification tasks. Deep learning models based on recurrent neural networks (RNN) and CNN models are also used for the de-identification of clinical notes [37]. The BioBERT [9], SciBERT [29], and recent Transformer-based models are also used to identify the named entities from biomedical texts.

In this work, we also use deep learning-based methods to build a pipeline for the biomedical NER and de-identification tasks. We identify many biomedical named entities including SDOH from COVID-19 texts.

## 3. Materials and Methods

### 3.1. Data Cohort

We have collected the scientific articles and clinical case reports from different journals (Lancet, BMJ, AMJ, Clinical Medicine and related) through LitCOVID [4] API, a resource of scholarly articles. The inclusion and exclusion criteria for data collection are given below:

- We specify the timeline between November 2021 and March 2022 for data collection.
- We specify English as the language to get the publications.
- We exclude many early-pandemic scientific articles, the intuition being that the disease symptoms and diagnosis, drugs and vaccination information were not clear during that time.
- We specify the population groups in adults: 19–44 years, middle-aged: 45–64 years, aged: 65+ years, during data collection.

After obtaining the scientific articles from these sources, we use the Spark OCR [38] library to automatically extract content from the PDF files and convert them into dataframes, where each row corresponds to one document (publication). After all these steps and filtration criteria, we acquired around 15 k scientific articles. Because we specify limited age groups in the population setting, English as the only language, and a time period of 5 months, the number of articles obtained here is lower than those obtained in the actual repository (LitCOVID) during that time period.

Gold-standard dataset: We annotated around 200 scientific articles from our collected dataset using the JohnSnowLabs annotation lab [39], and prepare a gold-standard dataset. A gold-standard dataset [40] means a corpus of text or a set of documents, annotated or tagged with the desired labels by expert annotators. We use the application of active learning [41] to re-annotate a larger portion of the data, where we specified the gold-standard data as the seed. By the end of this step, we acquired around 500 articles that were annotated. According to research [42], this amount of data is sufficient to begin training an NLP model. We used the following named entities, shown in Table 2, as the gold labels. We saved this data in CONLL [14] format.

### 3.2. Biomedical Named Entity Recognition Pipeline Structure

**Figure 1:**
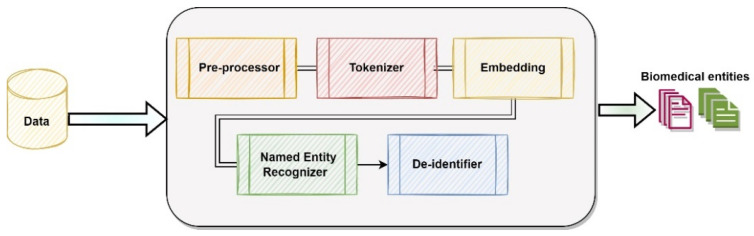
Biomedical Pipeline.

In this study, we propose a trainable ML pipeline that includes a pre-processor, tokenizer, embedding component, a deep neural network based on BiLSTM, CNN and CRF models, and a de-identifier. The novelty of this approach lies in the subtle integration of different components that are stacked together to train the pipeline. We build this pipeline following the Spark ML pipeline [43], which provides a default scalable solution without requiring much computation power [44]. The workflow of this pipeline is shown in Figure 1.

Next, we explain each component of this pipeline.

Data Collection: The input to the pipeline can be any raw textual data. We provided the data from our data cohort for this purpose.

Pre-processor: The preprocessor takes the text data as an input that comes from the data collection phase, pre-processes it, and detects the sentence boundaries in each record (document). Then, it transforms the data into a format that is readable by the next stage in the pipeline. The output from the pre-processor is the set of records that are pre-processed.

Tokenizer: The tokenizer takes the pre-processed data from the pre-processor as input. Tokenization is the process of breaking the input text into smaller chunks (words, or sentences) called tokens [45]. These tokens aid in comprehending the context and in developing the NLP model. The output from the tokenizer is transformed data, containing the tokens (words) corresponding to each document (scientific article, case report and so on).

Embedding: The tokenized data from the tokenizer goes into the embedding component, which maps tokens to vectors. We have fine-tuned the pre-trained BlueBERT model [46] that is trained on PubMed abstracts and MIMIC-III [47] on our gold-data to provide task-specific embeddings.

**Figure 2:**
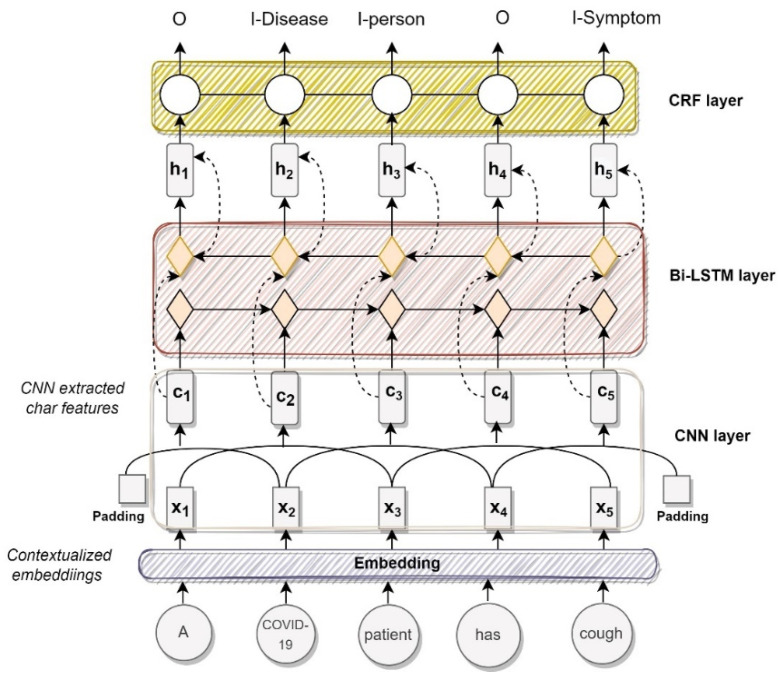
Named Entity Recognition algorithm.

Named Entity Recognizer: This component identifies biomedical entities in the text. This is an algorithm based on the BiLSTM-CNN-CRF [48] model. We modify the vanilla BiLSTM-CNN-CRF for the task-specific embeddings and make our modifications. We introduce our NER model in Figure 2 and explain its working below.

As shown in Figure 2, the algorithm takes as input the sequence of words or a sentence. This sequence is represented as $s=\left[w_{1},\;w_{2},\;\ldots ,\;w_{N}\right]$, where *N* is the sentence length and $w_{i}\;\in \;R^{V}$ is the $i_{th}$ token in the sequence. This input goes to the embedding layer.

The embedding layer is the first layer in the model that converts a sentence from a sequence of tokens into a sequence of dense vectors. In this work, we use our fine-tuned transformer model for the embeddings. The output of this layer is a sequence of vectors $x=\left[x_{1},\;x_{2},\;\ldots ,\;x_{N}\right]$, where $x_{i}=Ew_{i}\in \;R^{D}$, E is for embedding and $x_{i}$ is the dense vector representation of word $w_{i}$.

The second layer in this model is a CNN layer that is used to capture local information within given words in a biomedical context. The CNN is just for char embeddings to represent letters. The main feature is word embeddings coming from the embedding layer (BERT-based embeddings). The output of the CNN layer is $c=\left[c_{1},\;c_{2},\;\ldots ,\;c_{N}\right]$, where $c_{i}\in R^{M}$, *M* is the number of filters. The contextual representation $c_{i}$ of the $i_{th}$ character is the concatenation of the outputs of all filters at this position

The third layer in the model is the Bi-LSTM network, which is used to learn hidden representations of characters or tokens in a sequence using all of the previous contexts (in both directions). The output of the Bi-LSTM layer is $h=\left[h_{1},\;h_{2},\;\ldots ,\;h_{N}\right]$, where $h_{i}=R^{2S}$ and *S* is the dimension of hidden states in LSTM.

The fourth layer on the top of the Bi-LSTM network is the CRF layer [49]. The input to the CRF layer is the hidden representations of characters $h=\left[h_{1},\;h_{2},\;\ldots ,\;h_{N}\right]$ generated by the Bi-LSTM layer. To ensure that the predicted labels are valid, the CRF layer captures the dependency relationship between the named tags and constrains them to the final predicted labels [22]. The output of the CRF layer is $y=\left[y_{1},\;y_{2},\;\ldots ,\;y_{N}\right]$, which is a label sequence of sentence *s*, where $y_{i}\in R^{L}$ is the one-hot representation of the $i_{th}$ character’s label and L is the number of labels. In this work, the biomedical entities are the labels. A tanh layer on top of the BiLSTM layer is added to predict the confidence scores (CS) for the word with each of the possible labels as the output score of the network.

De-identifier: We use the data obfuscation technique, which is a process that obscures (masks) the meaning of data [50]. For example, to replace identified names with different fake names or to mask some data, value <02-02-2022> with <DATE> is used. This component provides HIPAA [13] compliance when dealing with text documents containing any protected health information. We use the pre-trained de-identification model from Johnsnowlabs [51] and embed it inside the pipeline to de-identify the personal records of the patients.

Biomedical Named Entities: The output of the pipeline is the biomedical entities, shown in Table 2.

### 3.3. Evaluation

We adopted a two-fold evaluation technique: (1) to evaluated the accuracy of the proposed approach, and (2) to analyze the results of our approach for pandemic surveillance. To evaluate the accuracy of the proposed approach, we considered a number of baseline methods and benchmark datasets including our test set. To evaluate the pandemic surveillance, we analyzed the results of our model and summarized the key findings.

Benchmark datasets: We used the JNLPBA [19] for chemical entities, NCBI-Disease [15] for disease entities, BC5CDR [16] dataset for chemical and disease mentions, BC2GM [18] for genes, and i2b2-Clinical [20] for clinical entities. From here, we obtained datasets that were already available in CoNLL-2003 format [52]. We performed further processing to convert them into IOB (Inside-Outside-Before) [53] scheme. All the datasets were divided into training, validation, and test sets, with a 70:15:15 ratio for all experiments. The Stratified 5-Folds cross-validation (CV) strategy was used for train/test split if original datasets did not have an official train/test split. We also set aside 30% of our gold dataset as a test set.

Baseline Methods: We compared the performance of our approach against the following state-of-the-art baseline methods: BiLSTM-CRF [54], BiLSTM-CRF-MTL [24], CT-BERT [55], SciBERT [29], and BioBERT [9] (v1.0, v1.1, v1.2). All of the baselines were trained on the aforementioned datasets. Each baseline was tuned to its optimal hyperparameter setting and the best results were reported for each method.

Training environment: All the experiments were run on Google Colab Pro (NVIDIA P100 or T4, 24 GB RAM, 2 x vCPU). The grid search was used to get optimal values for the hyperparameters and early stopping was performed to overcome overfitting. We specified the following hyperparameters as shown in Table 3.

Evaluation metrics: Following the standard practice [41,56] to evaluate NER tasks, we used the following metrics:

- Micro-average F1 to measures the F1-score of aggregated contributions of all classes.
- Macro-average F1that adds all the measures (Precision, Recall, or F-Measure) and divides with the number of labels, which is more like an average.

## 4. Results

The results and analysis are given below.

### 4.1. Comparison with Baseline Methods

We show the performance of our approach for accuracy in Table 4.

Overall, these results show that our approach achieved state-of-the-art performance on five public biomedical benchmarks, as well as on our dataset designed specifically for biomedical named entities. This demonstrates the generalizability of our methodology across different domains.

Our approach achieved the best micro F1 score of 94.78 on our dataset (52 entities), 90.58 on NCBI Disease (disease entity), 89.90 on BC5CDR (chemicals), 89.15 on BC2GM (gene/proteins), 79.92 on JNLBPA (chemical) and 89.10 on the i2b2 (clinical) dataset. We see similar patterns and higher performances in our pipeline for macro F1 scores.

The BioBERT model shows competitive performance in these results. Among the variants of the BioBERT, we see overall better performance of BioBERT v1.2 than its other variants, except for a few places, where BioBERT v1.1 marginally outperforms BioBERT v1.2. The better performance of BioBERT v1.2 is attributed to its training method, which is the same method as BioBERT v1.1 but includes an LM head [57]. Among the BERT-based models (BioBERT, SciBERT, CT-BERT), BioBERT performs best. The BioBERT is quite generalizable compared to other BERT-based methods, the SciBERT is initially trained on scientific data (not clinical) [29], and, CT-BERT is pre-trained on social media data, so they perform differently with different entity types.

Among the BiLSTM-based models (BiLSTM-CRF, BiLSTM-CRF-MTL), we observe the good performance of the BiLSTM-CRF model in identifying many diseases, chemicals, and gene/protein entities in these experiments. Our algorithm (BiLSTM-CNN-CRF) performs better than the BiLSTM-CRF baseline, probably because we are using biomedical embeddings on top of char-level embeddings. The fine-tuned transformer model’s embeddings enhance the performance of our model.

Although we fine-tuned each baseline method to its optimal hyperparameter settings, we anticipate that the relatively low scores of these baselines on our dataset can be attributed to the following: (i) the absence of an annotated dataset for training new biomedical entities, and (ii) different training/test set splits used in previous works that were unavailable.

Ablation Study: We performed an ablation experiment in which we evaluated the component of our pipeline. This component was based on our modified BiLSTM-CNN-CRF model. We replaced the standard BiLSTM-CNN-CRF in the sequence labeling architecture (Figure 2) with a direct feedforward map with and without a CRF decoder. We used a simple linear map over the embeddings to determine their direct information content. The results of this ablation study on our test set, based on macro average F1-score, are shown in Table 5.

The results, in Table 5, show that the effect of removing the BiLSTM layer is far more than removing the CRF layer from BiLSTM-CNN-CRF. This is shown with a dropped macro F1 of more than 15% when we remove the BiLSTM layer, compared to removing only the CRF layer. The most impacted performance is seen with Map-CNN where we removed these two layers (BiLSTM and CRF). With all these results, we find that our default settings are best in this setup.

### 4.2. Pandemic Surveillance

In this section, we demonstrate the effectiveness of our approach in demonstrating the key findings on pandemic surveillance. First, we show the most common entity types predicted by our approach after parsing 500 case reports, and show the performance of the model in terms of precision, recall, F1-score (F1), micro-average and macro-average in Table 6. The formulae for these performance metrics are based on true positives (TP), false positives (FP) and false negatives (FN).

**Figure 3:**
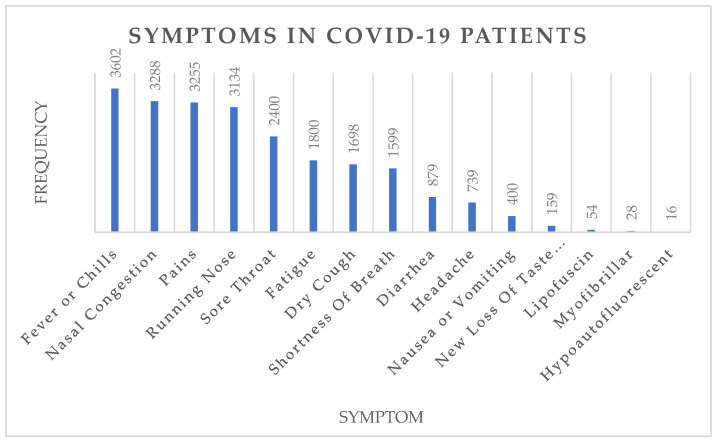
Most common symptoms of COVID-19 patients. Number at the top of each bar represents the number of times the symptoms were mentioned in test set.

As seen in Table 6, we can accurately predict a large number of entities with quite a high score. We also show the prevalence of the most common symptoms observed in our data in Figure 3.

The results in Figure 3 show that fever, nasal congestion, pains, a running nose, and sore throat are among the most common COVID-19 symptoms. Next, we show the most occurring named entities (occurrence > 70%) under the prominent entity types (drugs, vaccines, treatments) and show the results in Table 7.

We also gave a snippet from a COVID-19 related case report to our pipeline and show the confidence score for the predicted entities. The results are shown in Table 8.

The result in Table 8 shows that our model can predict many named entities with a high level of confidence score.

**Figure 4:**
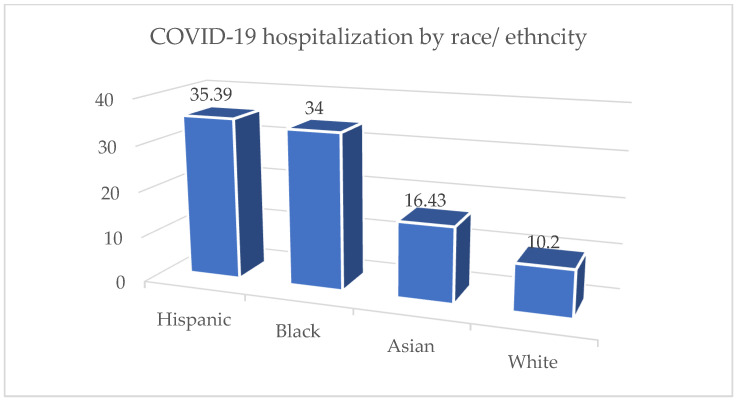
COVID-19 hospitalization by race and ethnicity.

We take the nominal race groups [58] and report the results where the race group accounts for more than 5% of the population. This finding shown in Figure 4 is based on a subset of available data from a specific time period, so it may not be an accurate representation of racial groups as a whole during the COVID-19 outbreak.

**Figure 5:**
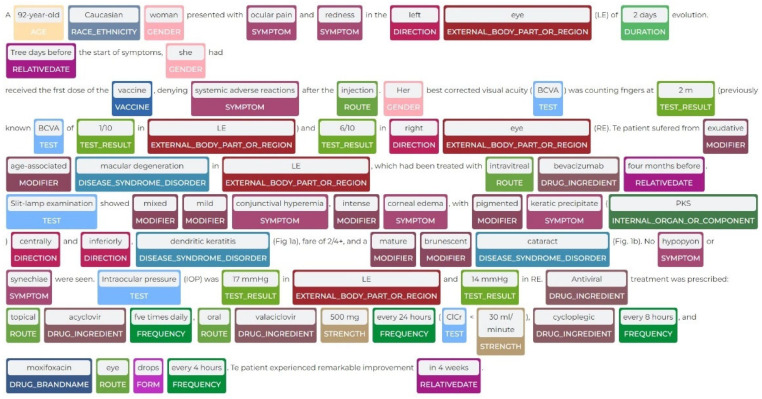
Biomedical entities recognized by proposed pipeline.

We show a sample prediction of our model on a case report [59] in Figure 5, where we can see that many clinical and SDOH are being detected.

## 5. Discussion

### 5.1. Implications in Healthcare

There are many different ways that this pipeline can be used in healthcare settings. These biomedical entity types can assist physicians, nurses, and other healthcare professionals in matching symptoms to a diagnosis, a course of treatment, and follow-up. Health disparities can be decreased by tracking social determinants [60]. The clinical data can be converted into knowledge, evidence, and clinical impact using this research as well. This pipeline emphasizes best practices, openness, reproducibility, automation, and the capacity to recognize complex named entities from biomedical texts. With little to no code modification, this pipeline can also be applied to any other domain.

### 5.2. Transfer Learning

The advantages of transfer learning in detecting COVID-19-named entities become clearer because of this work. The proposed approach (combining BiLSTM-CRF-CNN with Transformer-based embeddings) achieves a performance comparable to pure Transformer-based models (BioBERT), and performs at least 1 to 5% better compared to conventional BiLSTM models. In the future, it would be beneficial to have our own pre-trained embeddings that can be used to study a large number of clinical and non-clinical entities.

### 5.3. Limitations

Although the BiLSTM-CNN-CRF model that we used for this approach showed good results and outperformed the current state-of-the-art solutions, there is still room for improvement, and the following points are what we would consider implementing in the future: first, we plan to increase the number of layers in this deep neural network. We intend to pre-train a transformer-based model. In this regard, one approach would be to first prepare more data for annotation and then pre-train the model on the annotated data.

So far, we have annotated a portion of the dataset, which suffices for the purpose of model training. In the future, we strongly encourage the inclusion of medical professionals in the annotation guideline. We also plan to annotate a large number of documents for this type of study.

We also plan to test the model on additional benchmark datasets. Furthermore, we intend to curate more clinical data; in particular, getting real-time access to EHRs would be helpful. Since we are already providing a de-identifier to de-identify patients’ personal information through this pipeline, we hope to gain access to such a dataset soon while adhering to HIPAA guidelines. Lastly, due to the black-box nature of most deep neural networks, we also plan to handle bias or systematic error in research methods, which may influence disease associations and predictions.

## 6. Conclusions

In conclusion, this paper presents a pipeline that consists of a number of ML components stacked together. We used an approach to train models for the biomedical named entities using the BiLSTM-CNN-CRF model plus BERT-based embeddings. This paper shows that using contextualized word embedding, pre-trained on biomedical corpora, significantly improves the results of biomedical NER tasks. We evaluated the performance of this approach on benchmark datasets and our own test set, and our approach achieved the state-of-the-art results compared to the baselines. This pipeline can be used in different health science settings, provided that the annotated data to train the model and the pipeline is available.

## Figures and Tables

**Table 1:** Biomedical NER datasets and methods.

Benchmark Datasets		
Corpus	Entity Types	Data Size
NCBI-Disease [15]	Diseases	793 PubMed abstracts
BC5CDR [16]	Diseases	1500 PubMed articles
BC5CDR [16]	Chemicals	1500 PubMed articles
BC4CHEMD [17]	Chemicals	10,000 PubMed abstracts
BC2GM [18]	Gene/Proteins	20,000 sentences
JNLPBA [19]	Genes, proteins	2404 abstracts
i2b2-Clinical [20]	Problem, Treatment, and Test.	426 discharge summaries
I2b2 2012 [21]	Clinical (problems, tests, treatments, clinical departments, occurrences (admission, discharge) and evidence).	310 discharge summaries
**Benchmark methods**		
**Method**	**Description**	
BiLSTM-CRF [22]	Bidirectional Long short-term memory (LSTM) and Conditional random field (CRF) architecture for NER.	
BiLSTM-CNN-Char [23]	A hybrid LSTM and Convolutional Neural Network (CNN) architecture that learns both character-level and word-level features for the NER task.	
BiLSTM-CRF-MTL [24]	A multi-task learning (MTL) framework with a BiLSTM-CRF model to collectively use the training data of different types of entities.	
Att-BiLSTM-CRF [25],	Attention (Att) based BiLSTM model with a CRF layer for chemical NER task.	
Doc-Att-BiLSTM-CRF [26]	Document (Doc)-level Attention (Att)-based BiLSTM-CRF network for disease NER task.	
MCNN [27]	A multiple (M) label CNN-based network for disease NER from biomedical literature.	
CollaboNet [28]	A collaboration of deep neural networks, i.e., BiLSTM-CRF with a single task model trained for each specific entity type.	
SciBERT [29]	A pre-trained language model based on Bidirectional Encoder Representations from Transformers (BERT) pretrained on a large multi-domain corpus of scientific publications to improve performance on downstream scientific tasks including NER.	
BioBERT [30]	A pre-trained biomedical language representation model based on BERT for biomedical text mining	

**Table 2:** Biomedical entities used in this study.

Entity Type	Entities
Clinical name entities	Admission (patient admission status), oncology (tumor/cancer), blood pressure, respiration (e.g., shortness of breath), dosage (amount of medicine/drug taken), vital signs, symptoms, kidney disease, temperature (body), diabetes, vaccine, time (days, weeks or so), obesity (status), BMI, height (of patient), heart disease, pulse, hypertension, drug name, cerebrovascular disease, disease, treatment, clinical department, weight (of patient), admission/discharge (from hospital), modifier (modifies the current state), external body part, test, strength, route, test result.
Non-clinical entities	Name (of patient), location, date, relative date, duration, relationship status, social status, family history (family members, alone, with family, homeless), employment status, race/ethnicity, gender, social history, sexual orientation, diet (food type, nutrients, minerals), alcohol, smoking.

**Table 3:** Hyperparameters used—optimal parameter (range of values).

Hyperparameter	Optimal Value (Values Used)
Learning rate	1 × 10^−3^ (1 × 10^−2^, 1 × 10^−3^, 1 × 10^−5^, 2 × 10^−5^, 5 × 10^−5^, 3 × 10^−4^)
Batch size	64 (8, 16, 32, 64, 128)
Epochs	30 ({2, 3, …, 30})
LSTM state size	200 (200, 250)
Dropout rate	0.5 ({0.3, 0.35, …, 0.7})
Optimizer	Adam
CNN filters	2 (2, 3, 4, 5)
Hidden Size	768
Embedding Size	128
Max Seq Length	512
Warmup Steps	3000

**Table 4:** Test results using macro-average F1 (macro) and micro-average F1 (micro) scores on all datasets using different methods. The best scores are in bold and the second-best in italic.

Methods/ Dataset	Metric	NCBI	BC5CDR	BC2GM	JNLPBA	i2b2-Clinical	Our Dataset
BiLSTM-CRF	micro	85.80	84.22	78.46	74.29	83.66	87.10
	macro	86.12	85.09	80.01	75.10	84.01	*88.01*
BiLSTM-CRF-MTL	micro	86.46	84.94	80.34	77.03	82.38	88.39
	macro	88.01	85.00	81.12	77.14	83.96	88.97
CT-BERT	micro	77.50	76.85	74.10	68.00	77.07	78.10
	macro	78.50	77.96	75.37	68.98	78.01	78.98
SciBERT	micro	82.88	82.94	84.08	75.77	78.19	80.95
	macro	83.32	83.13	85.84	77.01	79.10	81.14
BioBERT-Base v1.0	micro	84.01	86.56	78.68	86.28	85.87	84.01
	macro	79.10	78.90	79.00	78.13	72.18	79.10
BioBERT-Base v1.1	micro	88.52	87.15	79.39	76.16	86.27	88.52
	macro	85.89	87.10	87.18	*75.45*	*87.78*	85.89
BioBERT-Base v1.2	micro	*89.12*	*87.81*	*83.34*	*76.45*	*86.88*	*89.12*
	macro	*86.78*	*87.89*	*86.07*	75.15	86.98	86.78
Our approach	micro	**90.58**	**89.90**	**89.15**	**79.92**	**89.10**	**94.78**
	macro	**91.83**	**90.34**	**90.38**	**80.94**	**90.48**	**95.37**

**Table 5:** Ablation study of the model. Bold shows best macro-average F1 score.

Model	Macro
BiLSTM-CNN-CRF	**94.18 ± 0.12**
BiLSTM-CNN	87.37 ± 0.02
Map-CNN-CRF	80.55 ± 0.03
Map-CNN	69.25 ± 0.04

**Table 6:** Performance of most used entity from random 500 case reports.

Entity	TP	FP	FN	Prec	Recall	F1
Disease	818	98	112	0.89	0.88	0.89
Gender	390	78	101	0.83	0.79	0.81
Employment	234	29	132	0.89	0.64	0.74
Race_Ethnicity	334	65	96	0.84	0.78	0.81
Smoking	309	24	97	0.93	0.76	0.84
Psychological_Condition	218	29	58	0.88	0.79	0.83
Death_Entity	387	34	103	0.92	0.79	0.85
BMI	146	12	29	0.92	0.83	0.88
Diabetes	157	10	28	0.94	0.85	0.89
**Macro-average**	2993	379	756	0.89	0.79	0.84
**Micro-average**	2993	379	756	0.89	0.80	0.84

**Table 7:** Most prevalent named entities under entity types (drugs, vaccine, treatments).

Drugs	Vaccine	Non-Medical Treatments
Hydroxychloroquine	Pfizer-BioNTech	Isolation
Paxlovid	Moderna	Wear masks
Actemra	AstraZeneca	Vaccination
Immunomodulators	CoronaVac	Oxygen support
Steroid	BBIBP-CorV	Medication
Amoxicillin	Janssen	Hand sanitization

**Table 8:** Test Results on all Datasets using different Methods.

Sentence	Begin	End	Chunks	Biomedical Entity	Confidence
0	2	12	73-year-old	Age	1.00
0	14	18	woman	Gender	1.00
0	32	43	Fever Clinic	Clinical Department	0.98
0	52	65	First Hospital	Clinical Department	0.51
0	109	134	Fever, temperature	Symptom	0.80
0	156	160	Cough	Symptom	0.99
0	163	175	Expectoration	Symptom	1.00
0	178	196	Shortness of breath	Symptom	0.39
0	203	218	General weakness	Symptom	0.77
0	233	244	Prior 5 days	Relative Date	0.42
1	247	249	She	Gender	1.00
1	261	264	Mild	Modifier	0.90
1	266	273	Diarrhea	Symptom	1.00
1	280	289	Stools/day	Symptom	0.85
1	292	303	2 days prior	Relative Date	0.68
1	322	329	Hospital	Clinical Department	1.00
1	386	402	COVID-19 positive	Disease Syndrome	0.90
1	436	454	Healthcare provider	Employment	0.94
2	486	494	Cirrhosis	Disease Syndrome	0.96
2	500	514	Type 2 diabetes	Diabetes	0.95
2	535	541	Smoking	Smoking	1.00
2	546	553	Drinking	Alcohol	0.93

## Data Availability

The data can be made available upon request from corresponding author.
